# Efficacy of Botulinum Toxin Type A for Treating Chronic Low Back Pain

**DOI:** 10.5812/kowsar.22287523.1845

**Published:** 2011-09-26

**Authors:** Seyed M. Jazayeri, Alireza Ashraf, Habib M. Fini, Hajar Karimian, Mohamadreza V. Nasab

**Affiliations:** 1 Department of Physical Medicine and Rehabilitation, Shiraz University of Medical Sciences, Shiraz, Iran; 2 Department of Physical Medicine and Rehabilitation, Shahid Sadughi University of Medical Sciences, Yazd, Iran

**Keywords:** Botulinum toxin type A, Low back pain, Pain

## Abstract

**Background:**

Low back pain is a major cause of disability and can result in substantial morbidity and high healthcare costs. Botulinum toxin has been used successfully to alleviate pain for a number of conditions caused by muscle contractions or spasms.

**Objectives:**

The aim of this study was to investigate the efficacy of botulinum toxin type A (BoNT-A; Dysport®, Ipsen, UK) for treating chronic low back pain (CLBP).

**Patients and Methods:**

This was a single-blind, randomized clinical trial study. Fifty patients with CLBP received either BoNT-A (40 Ipsen units per injection) or saline in 5 sites in the paraspinal muscles (n = 25 per group). A visual analogue system (VAS) was used to measure pain levels at baseline and at 4 and 8 weeks post-injection. Disability was assessed using the Oswestry low back pain disability questionnaire at baseline and at 8 weeks post-injection.

**Results:**

After 4 weeks, 76% of patients in the BoNT-A group reported pain relief compared to 20% in the saline group (P < 0. 005). Additionally, greater pain relief was experienced by patients in the BoNT-A group at 8 weeks (64% vs. 12%; P < 0. 001). By week 8, significant functional improvement (a minimum two-grade improvement between baseline and post-treatment assessments) was demonstrated in a higher number of patients receiving BoNT-A than in the saline group (68% vs. 12% , respectively; P < 0. 005). Patients experienced only minor side effects.

**Conclusions:**

BoNT-A improves CLBP with a low incidence of side effects and can be used as a therapeutic tool in the management of these patients.

## 1. Background

Back pain is a widespread cause of pain and disability and results in substantial healthcare costs worldwide (1). According to a report from the National Institute of Neurological Disorders and Stroke, “Americans spend at least $50 billion each year on low back pain, the most common cause of job-related disability and a leading contributor to missed work. ” Back pain is the second most common neurological ailment in the United States; only headache is more common (2). Although episodes of acute low back often resolve rapidly, they can become chronic, and approximately 30%-40% of cases result in persistent, disabling symptoms (either continuous pain or recurrent episodes) (3). Chronic low back pain (CLBP) impairs psychosocial, behavioral, vocational, and avocational measures of disability in many cultures and countries (4). CLBP can have a major detrimental effect on a patient’s quality of life and career since without adequate treatment, those with chronic back pain may be unable to work or perform daily tasks (5). Studies show that effective intervention in patients with low back pain may reduce morbidity and healthcare costs (6).

Chemical denervation using botulinum toxin has revolutionized treatment for many disorders involving excessive muscle activity (7), including movement disorders such as cervical dystonia (8, 9), tremor (10, 11), cerebral palsy (12-14), limb spasticity following stroke (15, 16), or multiple sclerosis (17-19). Botulinum toxin injections have also been reported to be beneficial in bringing about pain relief in a variety of conditions, including low back pain (20-24).

Ney et al. reported a significant improvement in back and radicular pain after botulinum type A toxin injection (24); in a study in Kuwait (63%), patients showed a remarkable recovery in visual analogue system (VAS) and functional state after botulinum type A toxin (BoNT-A) injection at 3 sites on either side of the lumbar paraspinal muscles (23).

## 2. Objectives

The aim of this study was to assess pain and disability reduction following local Clostridium BoNT-A hemagglutinin complex injections in paravertebral muscles at sites of maximum discomfort in CLBP patients. We investigated the minimal effective dose of toxin for safe outpatient treatment.

## 3. Patients and Methods

### 3. 1. Participants

A total of 50 patients with CLBP who were referred to our clinic over the course of 18 months were enrolled in this single-blind, randomized clinical trial. Patients who met the following inclusion criteria were randomized to receive either BoNT-A (Dysport®; Ipsen Ltd, Slough, UK) (n = 25) or saline (n = 25):

1. CLBP for a period of at least 6 months;
2. Ages 18–55 years old;
3. Consent to participate.

Exclusion criteria included the following: congenital or severe spine deformity; chronic systemic disorder; history of allergic reaction to BoNT-A; current or planned pregnancy; breast feeding; history of neuromuscular junction disorder; presence of motor neuron disease; primary muscle weakness; malignancy; bleeding tendency, as well as the presence of acute pathology; any previous injection in paravertebral muscles in a 6-month period; and suspected secondary gain. At each session, patients were evaluated for current and past medical history and given a physical examination.

### 3. 2. Study Treatment

BoNT-A was prepared by re-constituting frozen-dried toxin with sodium chloride injection BP to a concentration of 100 Ipsen units/mL. The solution was drawn into a 1-mL syringe and injected through a 25-gauge needle. The needle size was adjusted according to the patient’s level of body fat to ensure that the study treatment was delivered to the target injection site. Patients were given 5 injections of 40 Ipsen units each at 5 equidistant lumbosacral sites (L1 to S1) in the paraspinal muscles. Tender or trigger points were chosen as starting points for injections in both groups. All patients were injected by the same person once unilaterally on the side of maximum discomfort. During the study, patients were recommended to continue previously prescribed medication at their normal dosages.

### 3. 3. Assessment Measures

The level of pain was evaluated in each patient using the VAS (25). The degree of physical impairment and disability were assessed using the Oswestry Low Back Pain Disability Questionnaire (OLBPQ) (26). Both assessment measures have been shown to be reliable measures of pain and disability in patients with low pain (25, 27). The OLBPQ is a questionnaire designed to assess the functional ability of patients to carry out daily living tasks. It consists of 10 subsets, including questions regarding pain intensity, personal care, lifting, walking, sitting, standing, sleeping, sexual activity, social life, and travel. Each subset is graded from 0 (normal) to 5 (most affected). Significant functional improvement on the OLBPQ was defined as at least a two-grade improvement between baseline and post-treatment assessments in 4 or more functional subsets. Subjects completed questionnaires at baseline and at 8 weeks post-treatment.

The VAS consisted of a 10-cm horizontal line, labeled with “no low back pain” at one end and “worst low back pain” at the other end. Patients were asked to mark the line at a point on the line corresponding to the level of pain they typically experienced. The distance between the point of “no low back pain” and the patient’s mark was measured in centimeters and used as a numeric index of pain severity and change in pain. A significant clinical improvement in pain relief was defined as a 50% or greater difference in pre- and post-treatment VAS scores. VAS assessments were recorded at baseline and at 4 and 8 weeks post-treatment.

### 3. 4. Statistical Analysis

Results were analyzed by an independent data management group. SPSS software version 15 was used for statistical analysis (Cary, NC, USA). Patients were included in analysis on the basis of intention-to-treat. The VAS score at baseline, 4 weeks, and 8 weeks was tested using repeated measures analysis of variance (ANOVA). The normality of variables was evaluated using Kolmogorov–Smirnov tests. According to the normality of the variable, the 2-sided paired and t-test (Wilcoxon signed-rank test) were used for between- and within-group comparisons, respectively. Demographic variables were compared using Fisher’s exact test and t-test. Adverse event frequencies were compared using the chi-square and Fisher’s exact tests.

## 4. Results

### 4. 1. Demographic Data

All 50 patients consented and completed the study. The mean age of patients was 42 years (range, 18–55 years). Of the 25 patients in each group, 13 women were assigned to the BoNT-A group and 14 women received saline. The mean duration of pain was 4 years (8 months to 9 years) and the mean pain score on the VAS was 6. 5. At baseline, functional impairment was recorded in 4 to 10 subsets on the OLBPQ. On the basis of ANOVA results (age, sex, and duration of pain), we concluded that there was no significant differences between the treatment and control groups. A comparison of both groups showed no baseline differences in VAS score and OLBPQ subsets (Table 1).

**Table 1.** Demographic Data of Patients Receiving BoNT-A or Saline

	BoNT-A ^a^	Saline
Number of patients	25	25
Men:Women	12:13	11:14
Mean (range), y	41. 7 (21-55)	42. 3 (18-53)
Mean pain duration, y	4. 4	3. 6
Baseline VAS^a^ score	6. 1	6. 9

^a^Abbreviations: BoNT-A, Botulinum toxin type A; VAS, Visual analogue system

### 4. 2. Pain Relief

At week 4, 19 of 25 patients (76%) who received BoNT-A showed a clinical response on the VAS compared with 5 of 25 patients (20%) in the group receiving saline (P < 0. 005 BoNT-A vs. saline). At week 8, 16 of 25 patients (64%) in the BoNT-A group experienced significant pain relief compared with 3 of 25 patients (12%) in placebo group (P <0.011 BoNT-A vs. saline).

### 4. 3. Functional Improvement

Functional improvement was also demonstrated in patients who received BoNT-A (Table 2). By week 8, 17 of 25 patients (68%) in the BoNT-A group demonstrated clinical improvement compared with 3 of 25 patients (12%) receiving saline (P < 0. 005).

**Table 2.** Improvements in Pain and Function Disability

Pain Relief (VAS) ^a^	BoNT-A ^a, b^ (n = 25), No. (%)	Saline (n = 25), No. (%)	*P* value
Week 4	19 (76)	5 (20)	< 0. 005
Week 8	16 (64)	3 (12)	< 0. 011
Functional improvement (OLBPQ) ^a, c^			
Week 8	17 (68)	3(12)	< 0. 005

^a^Abbreviations: BoNT-A, Botulinum toxin type A; VAS, Visual analogue system

^b^Defined as a 50% or greater difference in pre- and post-treatment VAS scores

^c^Defined as at least a two-grade improvement between baseline and post-treatment assessments in 4 or more functional subsets on the OLBPQ

### 4. 4. Safety Profile

There was no evidence of significant complications or pain exacerbation in either group.

## 5. Discussion

The aim of this study was to assess pain and disability reduction following local Clostridium botulinum type A toxin injections and placebo treatment in patients with CLBP.

In this single-blind, placebo-controlled study, significant inter-group differences for individual pain scores were demonstrated. Patients who received BoNT-A as a therapeutic intervention showed a lower pain score compared to the placebo group. Furthermore, our study showed a statistically significant difference in functional improvement in the BoNT-A-receiving group. Treatment was safe, and no side effects were observed. Our results are similar to those of a small, double-blind trial involving BoNT-A administration for treating CLBP (21). Similarly, Jabbari et al. , in an open label study involving 75 patients, reported a significant reduction in low back pain after BoNT-A injection (28). Subinetal, in a smaller randomized trial involving 19 patients with low back pain who were followed-up at 1 and 6 months, showed improvement in McGill Scores and Oswestry and Roland-Morris scores (29). Ney et al. reported a significant improvement in back and radicular pain after injection of BoNT-A (24).

BoNT-A is a potent inhibitor of acetylcholine release as well as a number of other neurotransmitters and neuropeptides. Botulinum toxin exerts its effects through inhibiting excessive acetylcholine release; it also has muscle relaxant properties, preventing stimulation of muscle nociceptors. It has also been postulated that botulinum toxin may have an analgesic effect, although the precise mechanism of action is unknown (30, 31). Additionally, in vitro inhibition of substance P by BoNT-A has been reported (32). Our study indicates the benefits and safety of paraspinal administration of botulinum toxin A for low back pain that has been demonstrated in previous studies. There are a few limitations to our study. First, our study evaluated a small number of patients. Furthermore, the extent of patient and physician bias is unclear. Patients may have over- or underestimated the effect of the medication based on preconceived expectations. Administration of BoNT-A to reduce muscle tone and over-activity that has occurred through injury and inflammation should form part of an overall treatment approach that also includes physical therapy. Ultimately, restoration of both normal muscle length and biomechanical balance will benefit patients by improving long-term pain relief. Further investigations of BoNT-A in chronic back pain are necessary to determine the reproducibility of the results and the long-term effect of repeated injections. Consideration should also be given regarding BoNT-A dose, the number and site of injections, the necessity of follow-up injections, and the length of treatment appropriate to the disorder. In patients with CLBP, 200 Ipsen units of BoNT-A administered at 40 Ipsen units over 5 sites was effective in conferring pain relief and showed no side effects.
